# Q221K mutation in VP2 drives antigenic shift of infectious bursal disease virus

**DOI:** 10.3389/fimmu.2025.1600371

**Published:** 2025-07-10

**Authors:** Haifeng Xiong, Jiayan Wu, Quan Xie, Tuofan Li, Zhimin Wan, Aijian Qin, Jianqiang Ye, Hongxia Shao

**Affiliations:** ^1^ Key Laboratory of Jiangsu Preventive Veterinary Medicine, Key Laboratory for Avian Preventive Medicine, Ministry of Education, College of Veterinary Medicine, Yangzhou University, Yangzhou, Jiangsu, China; ^2^ Jiangsu Co-Innovation Center for Prevention and Control of Important Animal Infectious Diseases and Zoonoses, Yangzhou University, Yangzhou, Jiangsu, China; ^3^ Joint International Research Laboratory of Agriculture and Agri-Product Safety, The Ministry of Education of China, Yangzhou University, Yangzhou, Jiangsu, China; ^4^ Institutes of Agricultural Science and Technology Development, Yangzhou University, Yangzhou, Jiangsu, China

**Keywords:** nVarIBDV, VP2, mAb, 221K, antigenicity, differentiation

## Abstract

**Introduction:**

Infectious bursal disease (IBD) is a severe immunosuppressive disease caused by the infection of infectious bursal disease virus (IBDV) in chicken. Recently, an emerging mutant named novel variant IBDV (nVarIBDV) has rapidly spread in China and become a prevalent strain. However, little is known about the unique antigenic sites of nVarIBDV escaped from current IBDV vaccines.

**Methods:**

Here, the expressed hypervariable region (HVR) of VP2 (VP2-HVR) of nVarIBDV was used as an immunogen and a novel monoclonal antibody (mAb) against VP2 (mAb 5B5) was generated.

**Results:**

Immunofluorescence assay (IFA) and ELISA demonstrated that mAb 5B5 specifically reacted with nVarIBDV and its VP2 protein, but not with classical IBDV (cIBDV), very virulent IBDV (vvIBDV), or attenuated IBDV (attIBDV) strains. Epitope mapping and site mutagenesis assay revealed that mAb 5B5 recognized the conformational epitope in peak A (212–224 aa) and heptapeptide (326–332 aa) regions, and identified residue 221K in VP2 as the key antigenic site, which is conserved exclusively in nVarIBDV strains. Notably, K221Q mutation in VP2 of nVarIBDV significantly altered the reaction profile for sera against vvIBDV or cIBDV. Neutralization assays revealed that mAb 5B5 could inhibit replication of an engineered attIBDV carrying 221K in Leghorn male hepatoma (LMH) cells. Structural analysis further found that 221K is surface-exposed and alters local electrostatic potential, possibly facilitating immune evasion.

**Discussion:**

All these demonstrated that 221K is a unique antigenic site in VP2 of nVarIBDV associated with immune escape, providing novel insights into the antigenicity of nVarIBDV and novel targets for efficient diagnostics, vaccine design, and molecular surveillance of IBDV.

## 1 Introduction

Infectious bursal disease (IBD) is an acute and highly contagious infectious disease caused by the infectious bursal disease virus (IBDV). IBDV infection in chickens aged 3–6 weeks generally results in severe immunosuppression (1, 2). IBDV is an icosahedral, non-enveloped and double-stranded RNA virus with two segments (A and B), which belong to the genus *Avianbirnavirus* of the family *Birnaviridae* (3). Segment A (3.2 kb) encodes viral proteins VP2, VP3, VP4 and VP5, whereas segment B (2.7 kb) only encodes protein VP1. Among these proteins, VP2 is the unique external capsid protein and the main protective antigen of IBDV (4). The IBDV crystal structure revealed that VP2 is folded into three domains, including base (B), shell (S), and projection (P) (5–7). B and S domains are relatively conserved, consisting of N- and C-terminal stretches of VP2, while the P domain is highly variable, consisting of the hypervariable region (HVR) of VP2 (VP2-HVR). VP2-HVR contains four hydrophilic regions (peak A (210–225 aa), minor peak 1 (247–254 aa), minor peak 2 (281–292 aa), and peak B (312–324 aa)) which include loop P_BC_, loop P_DE_, loop P_FG_ and loop P_HI_, as well as one extra heptapeptide region (326–332 aa). VP2-HVR plays vital roles in cell tropism, virulence and antigenic variation of IBDV (4, 8–10).

Based on cross-neutralization assays, IBDV has two distinct serotypes (I and II). Generally, serotype I, but not serotype II, is pathogenic to chickens. Serotype I IBDV exhibits significant genetic diversity and is traditionally classified into four major phenotypes based on pathotypes and antigenicity: classical IBDV (cIBDV), variant IBDV (varIBDV), very virulent IBDV (vvIBDV) and attenuated IBDV (attIBDV) (11). In 1957, cIBDV (classical IBDV) was first isolated in the United States. Within the subsequent four decades, varIBDV (variant IBDV) and vvIBDV (very virulent IBDV) emerged successively. vvIBDV, with higher lethality and transmissibility, rapidly spread worldwide. China first isolated cIBDV in 1979 (12), followed by the isolation of vvIBDV and varIBDV (13) in 1991 and 1996, respectively. The evolution of segmented dsRNA IBDV is driven by gene mutation, segment reassortment, and homologous recombination, which result in the generation of novel variant, reassortant, and recombinant IBDV strains (14–18). Widespread IBDV vaccine usage facilitated the persistent prevalence of vvIBDV and HLJ0504-like vvIBDV in China before 2016, making them focal points for prevention and control (16, 18–22).

Since 2016, increasing reports of suspected subclinical IBDV infections have emerged across China (23). A novel variant IBDV (nVarIBDV) was subsequently isolated in China and has rapidly spread to countries including Korea, Egypt, Malaysia, and Japan (24–27). Due to the increasingly complex molecular characteristics arising from cumulative mutations in emerging variants, traditional pathotype- and antigenicity-based classifications fail to define novel IBDV variants. To address this limitation, Michel and Jackwood (28) proposed a classification scheme of IBDV into seven genogroups based on VP2-HVR. However, both segments A and B are implicated in pathotype and genetic evolution of IBDV. Recently, an improved classification for IBDV based on both segments was proposed (29, 30), resulting in nine genogroups of A (A1, classical; A2, US antigenic variant; A3, very virulent; A4, dIBDV; A5, atypical Mexican; A6, atypical Italian; A7, early Australian; A8, Australian variant and A0, serotype 2) and five genogroups of B (B1, classical-like; B2, very virulent-like; B3, early Australian-like; B4, Polish & Tanzanian and B5, Nigerian). Under this scheme (30), the cIBDV, varIBDV, vvIBDV, and attIBDV correspond to genotypes A1B1, A2B1 (including A2aB1, A2bB1, and A2cB1), A3B2, and A8B1, respectively.

Global IBDV genetic diversity continues to expand. Following the identification of genotype A2dB1b nVarIBDV in China (30), a novel IBDV genotype (A9B1) was subsequently detected by Portuguese investigators (31). Both genotypes exhibit unique VP2 antigenic site variations, indicating potential challenges to current vaccine efficacy. Circulating Chinese IBDVs comprise genotypes A1B1 (cIBDV), A2dB1 (nVarIBDV), A3B2 (vvIBDV)/A3B3 (HLJ0504-like vvIBDV), and A8B1 (attIBDV). Additionally, a novel reassortant strain (genotype A2dB3) was recently identified in China, featuring segment A from nVarIBDV (A2dB1) and segment B from an HLJ0504-like vvIBDV (A3B3) (32, 33). Although nVarIBDV (A2dB1) generally causes subclinical signs without significant morbidity or mortality, nVarIBDV has become one of the prevalent types together with vvIBDV (A3B2/A3B3), resulting in severe immunosuppression in infected chickens with huge economic losses to poultry industry and posing significant challenges for prevention, diagnosis and control of IBD (2, 20, 34). Recent study reports that nVarIBDV exhibits significantly different antigenicity from vvIBDV (35), and vvIBDV vaccines cannot provide effective protection against nVarIBDV infection (36). However, little is known about the unique antigenic sites of nVarIBDV different from other IBDV types. In this study, we used the expressed VP2-HVR as an immunogen to generate a novel monoclonal antibody (mAb) against VP2 (mAb 5B5), and identified a unique antigenic site 221K in VP2 protein of nVarIBDV, which drives antigenic shift of IBDV.

## 2 Materials and methods

### 2.1 Viruses, cells and antibodies

Five IBDV strains—G61 (cIBDV, A1), 2340 and 2331 (nVarIBDV, A2d), 2341 (vvIBDV, A3), and NF8 (attIBDV, A8)—were isolated and preserved in our laboratory. NF8 is an attIBDV strain and kept in our laboratory. Leghorn male hepatoma (LMH) cells from ATCC were cultured in Dulbecco’s modified Eagle medium F12 (DMEM/F12) (Gibco, NY, USA) supplemented with 10% fetal bovine serum (FBS) (Lonsera, Shanghai, China), 100 U/mL penicillin and 100 µg/mL streptomycin at 37°C and 5% CO2 atmosphere. SP2/0 myeloma cells from our laboratory were maintained in HT medium. The monoclonal antibodies mAb 6C5 against VP2 protein of IBDV and mAb 2G10 (37) against the Fiber-2 protein of Duck adenovirus 3 (DAdV-3) were generated and stored in our laboratory. Chicken sera against nVarIBDV, vvIBDV or cIBDV were prepared and stored in our laboratory.

### 2.2 Primers, plasmids and animals

All primers used in the experiments are listed in **Table 1**. All eukaryotic recombinant plasmids were constructed into the pcDNA3.1 vector via homologous recombination, each fused with a C-terminal Flag tag. These recombinant plasmids include pc-nVarIBDV-VP2, pc-vvIBDV-VP2, pc-attIBDV-VP2, pc-cIBDV-VP2, pc-VP2-HVR, pc-201–347 aa, pc-201–327 aa, pc-201–307 aa, pc-201–287 aa, pc-201–267 aa, pc-201–337 aa, pc-225–337 aa, pc-VP2-delA, pc-VP2-delhep, pc-nVarIBDV-N213D, pc-nVarIBDV-K221Q, pc-nVarIBDV-K249Q, pc-nVarIBDV-I252V, pc-nVarIBDV-N254G, pc-nVarIBDV-D318G, pc-nVarIBDV-E323D, pc-nVarIBDV-K221Q, pc-vvIBDV-Q221K, pc-cIBDV-Q221K, pc-attIBDV-Q221K. BALB/c mice were purchased from experimental center for comparative medicine, Yangzhou University.

**Table 1:** PCR primers used for epitope mapping.

Primer	Direction	Primer sequence (5’ - 3’)
201aa-F	Forward	GATTACAAGGACGACGATGATGAGGCCCAGAGTCTAC
359aa-R	Reverse	GTCGTCCTTGTAATCTGTTGCCACTCGTTC
347aa-R	Reverse	GTCGTCCTTGTAATCGAGGGCTCCTGGATA
327aa-R	Reverse	GTCGTCCTTGTAATCCGACATCTGTTCCCCTG
307aa-R	Reverse	GTCGTCCTTGTAATCTGTGATTGGCTGGGTTA
287aa-R	Reverse	GTCGTCCTTGTAATCGATGCCGGCCGTCAGCC
267aa-R	Reverse	GTCGTCCTTGTAATCAAAGCCTATAAGGTAG
delpeakA-VP2-F	Forward	CTGCAGCCGTAACAATCACACTGTTCTCAG
delpeakA-VP2-R	Reverse	TTGTTACGGCTGCAGTTATGGTGTAGACTC
delhep-VP2-F	Forward	GAACAGATGCTAGCGGTGACGATCCATGGT
delhep-VP2-R	Reverse	CCGCTAGCATCTGTTCCCCTGTCTGGCCAT
N213D-F	Forward	GATGATTACCAATTCTCATCACAGTACAAG
N213D-R	Reverse	GAATTGGTAATCATCGGCTGCAGTTATGGT
D318G-F	Forward	AAAAGTGGCGGCCAGGCAGGGGAACAGATG
D318G-R	Reverse	CTGGCCGCCACTTTTGGAGGTCACTATCTC
E323D-F	Forward	GCAGGGGATCAGATGTCGTGGTCGGCAAGT
E323D-R	Reverse	CATCTGATCCCCTGCCTGGCCATCACTTTT
nVarIBDV-K221Q-F	Forward	CAGTACCAGACAGGTGGGGTAACAATCACA
nVarIBDV-K221Q-R	Reverse	ACCTGTCTGGTACTGTGATGAGAATTGGTA
vvIBDV-Q221K-F	Forward	AGTACAAGACAGGTGGAGTTACAATCACAC
vvIBDV-Q221K-R	Reverse	ACCTGTCTTGTACTGTGATGAGAATTGGTA
cIBDV-Q221K-F	Forward	AGTACAAGCCAGGTGGGGTAACAATCACAC
cIBDV-Q221K-R	Reverse	CCTGGCTTGTACTGTGATAAGAATTGGTAA
attIBDV-Q221K-F	Forward	AGTACAAGTTAGGTGGGGTAACAATCACAC
attIBDV-Q221K-R	Reverse	CCTAACTTGTACTGTGATGAGAATTGGTAA
K249Q-F	Forward	TGTTCCAAACCAACATCCAAAACCTTGTAC
K249Q-R	Reverse	TGTTGGTTTGGAACACAAGCTCCCCCCCAA
I252V-F	Forward	CCAACGTACAAAACCTTGTACTGGGCGCCA
I252V-R	Reverse	GGTTTTGTACGTTGGTTTTGAACACAAGCT
N254G-F	Forward	TCCAAGGCCTTGTACTGGGCGCCACTATCT
N254G-R	Reverse	GTACAAGGCCTTGGATGTTGGTTTTGAACA

### 2.3 Expression of VP2-HVR protein of nVarIBDV

The VP2-HVR fragment was amplified from the nVarIBDV cDNA template using the primers pColdI-VP2-HVR-F/R and then cloned to pColdI vector. The correct recombinant plasmid named pColdI-VP2-HVR was transformed into *E. coli* Rosetta (DE3) cells and the expression of VP2-HVR was induced with 1.0 mM isopropyl β-D-thiogalactoside (IPTG) at 16°C for 15 h. Then cells were collected, resuspended in PBS, lysed by sonication and centrifuged. The supernatants and precipitates were collected for protein identification by SDS-PAGE and Western blot. The expressed VP2-HVR protein was then purified by Ni-Sepharose high-performance affinity gel (GE Healthcare Life sciences, Freiburg, Germany) according to manufacturer’s instructions and identified by SDS-PAGE and Western blot analysis.

### 2.4 Generation of monoclonal antibodies

The purified VP2-HVR protein was mixed with Freund’s complete adjuvant (Sigma-Aldrich, Missoula, MO, USA), and then was intraperitoneally injected into BALB/c mice at a dose of 50 μg/mouse for the first immunization. The second and third immunization were performed with 50 μg purified VP2-HVR protein mixed with Freund’s incomplete adjuvant (Sigma-Aldrich, Missoula, MO, USA) every two weeks. Mouse with the highest serum titers against VP2 was selected and immunized with 50 μg of VP2-HVR protein by intraperitoneal injection without adjuvant. Three days later, the spleen cells from the immunized mice were fused with SP2/0 cells according to the procedure previously described (38). To screen positive hybridoma cells, LMH cells transfected with the pc-VP2-HVR were used as antigen in IFA. Then the positive hybridomas were subcloned for three times by limiting dilution method. The ascites of the corresponding positive hybridomas were prepared using paraffin-primed Balb/c mice, and purified by the NAb™ Spin Columns (Thermo scientific™). The subclass of mAbs was identified using the mouse monoclonal antibody subtype identification Kit (Biodragon, Beijing, China).

### 2.5 IFA

LMH cells transfected with serial truncations of VP2 protein (pc-201–347 aa/201–327 aa/201–307 aa/201–287 aa/201–267 aa/201–337 aa/225–337 aa, pc-VP2-delA and pc-VP2-delhep) or VP2 proteins from IBDV strains of different virulence (pc-nVarIBDV-VP2, pc-vvIBDV-VP2, pc-attIBDV-VP2 and pc-cIBDV-VP2) were fixed with chilled acetone: ethanol solution (3:2) for 5 min. Then the solution was discarded and the fixed LMH cells were dried for 1 h. Subsequently, mAbs 5B5 or Flag, and IBDV antisera were used as the primary antibody, while the FITC-labeled goat anti-mouse or anti-chicken IgG (Sigma-Aldrich, USA) served as the secondary antibody. Both were diluted with PBS and then incubated with LMH cells at 37°C for 45 minutes. After three washes with PBS, LMH cells were observed under an inverted fluorescence microscope. The fluorescence intensity was analyzed using ImageJ (NIH). Briefly, the images were converted to grayscale, followed by setting the threshold and measurement area (with fixed threshold and range). The relative fluorescence intensity was quantified using the formula: (Mean fluorescence intensity of test group [IntDen/Area])/(Mean fluorescence intensity of positive group).

### 2.6 Western blot

LMH cells transfected with indicated plasmids or infected with attIBDV were lysed with RIPA buffer (CWbio, Beijing, China) containing protease and phosphatase inhibitors (CST, MA, USA) for 20 min on the ice. The supernatant of the lysate collected by centrifuging and mixed with SDS loading buffer was boiled and then used for SDS-PAGE analysis. Subsequently, the proteins were transferred onto nitrocellulose membrane (GE Healthcare Life sciences, Freiburg, Germany). The membrane was then blocked with 5% skim milk in PBST and incubated with mAbs 5B5 or 6C5 at room temperature for 2 h. After being washed three times with PBST, the membrane was incubated with HRP-labeled goat anti-mouse IgG, for 1 h. Following additional three washes with PBST, the membrane was developed with luminol and peroxide (CWbio, Beijing, China) and imaged using an automatic imaging system (Tanon 5200). Densitometric analysis of Western blot bands was conducted using ImageJ (NIH). The analytical procedure included background subtraction correction, where identically sized measurement frames were applied to capture both target band signals and adjacent background signals in band-free regions, with background values subsequently deducted from target values. All data were normalized to GAPDH as an internal control, with relative expression levels calculated as the ratio of corrected target protein density to corresponding GAPDH density values.

### 2.7 Sandwich ELISA

The sandwich ELISA was used to verify the cross-reactivity of monoclonal antibodies with different types of IBDV. Briefly, the purified mAb 6C5 was diluted with a coating buffer (0.159 g Na_2_CO_3_, 0.293 g NaHCO_3_, 100 mL ddH_2_O, pH 9.6) to a final concentration of 15 μg/mL, and coated in ELISA plate with 100 μL per well at 4°C overnight. After three times washing with PBST buffer (PBS containing 0.05% Tween-20, pH 7.4), the wells were blocked with 5% skim milk at 37°C for 2 h. Washing three times as above, 100 μL dilution of different types of IBDV (nVarIBDV, vvIBDV, cIBDV and attIBDV) with the same viral copy number was added and incubated at 37°C for 1 h. After washing, 100 μL HRP conjugated mAbs 5B5, 6C5 and 2G10, diluted 1:300, were added and incubated for 1 h. Following another three washes, the 100 μL volume of 3, 3’,5,5’-Tetramethylbenzidine (TMB) ELISA substrate solution (Solarbio, Beijing, China) was added and incubated at 37°C for 15 min in the dark. Finally, 50 μL of 2 M H_2_SO_4_ was added to terminate the reaction and the values of OD_450_ nm were measured using a Microplate Spectrophotometer.

### 2.8 Epitopes mapping

**Figure 1:**
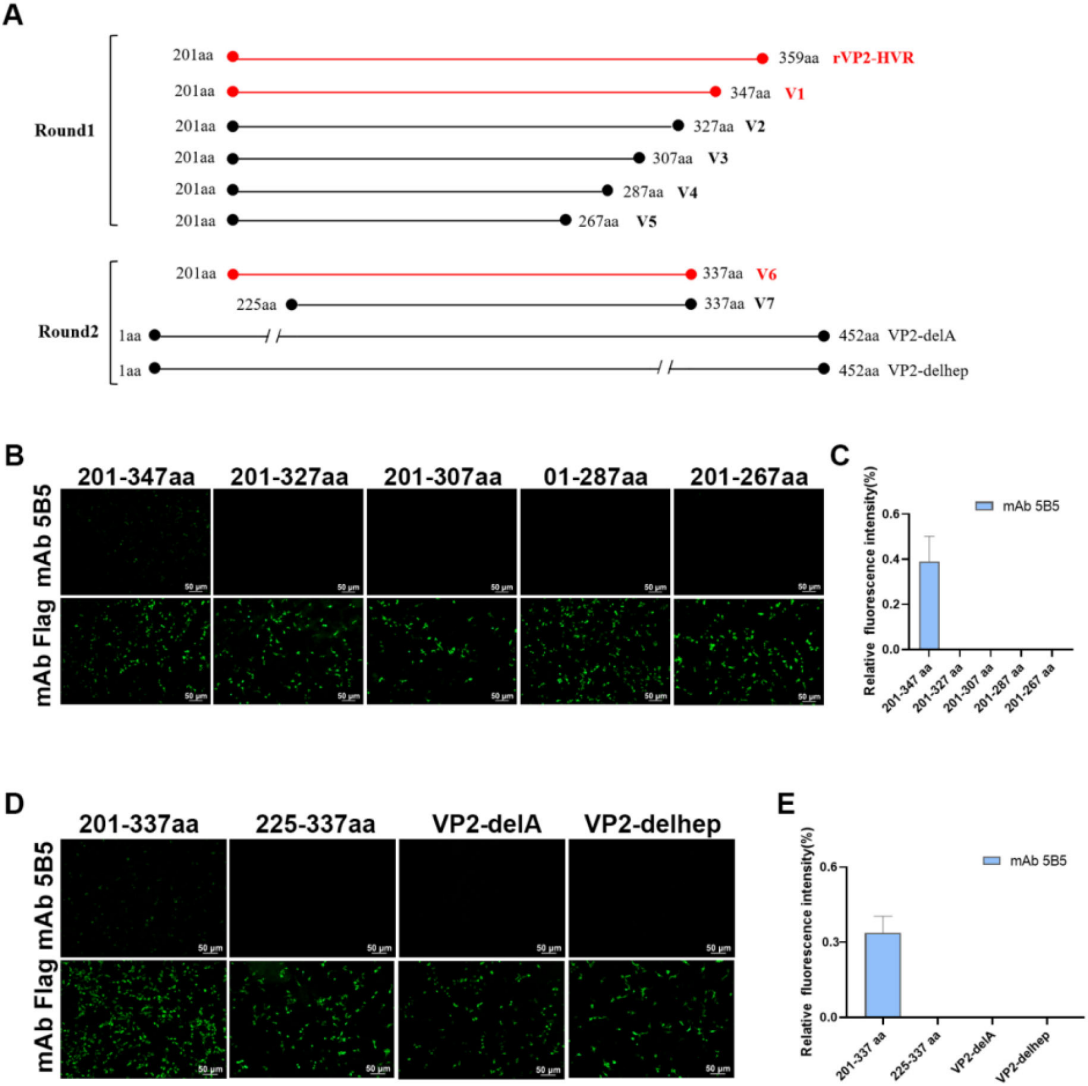
Both peak A and heptapeptide region are critical conformational epitopes recognized by mAb 5B5. A series of truncated VP2-HVR constructs tagged with Flag were generated and transfected into LMH cells. At 48 h after transfection, the cells were fixed for IFA. **(A)** Schematic representation of truncated VP2 fragments used for epitope mapping. **(B, D)** IFA analysis of epitopes of mAb 5B5 using VP2-HVR truncation variants and VP2 deletion mutants. **(C, E)** Quantification of fluorescence signals corresponding to **Figures 2B, D**. The percentage of mAb 5B5-bound positive cells (green fluorescence) to mAb Flag-bound positive cells (green fluorescence) was showed by using ImageJ (NIH).

To map the epitope recognized by mAb 5B5, a series of truncated VP2-HVR variants were cloned into the pcDNA3.1 vector. During the first screening round, five truncated constructs spanning residues 201–347 aa (V1), 201–327 aa (V2), 201–307 aa (V3), 201–287 aa (V4), and 201–267 aa (V5) were expressed. In the second round, V1 was further dissected into two overlapping peptides (201–337 aa and 225–337 aa). Additionally, using pc nVarIBDV-VP2 as the template, deletion mutants lacking either the peak A region (Δ212–224 aa) or the heptapeptide motif (Δ326–332 aa) were generated, respectively. The truncation strategy is schematically illustrated in **Figure 1A**. Furthermore, site-directed mutagenesis was performed to introduce single amino acid substitutions (N213D, K221Q, K249Q, I252V, N254G, D318G, E323D) into pc-nVarIBDV-VP2, and reciprocal mutations (Q221K) were engineered into pc-cIBDV-VP2, pc-vvIBDV-VP2, and pc-attIBDV-VP2. Epitope mapping was subsequently conducted using the IFA described above.

### 2.9 Rescuing of IBDV with point mutation

The infectious clone of the NF8 strain (attIBDV) was constructed as previously described (39). All primers involved below are from the **Table 2**. The infectious clone of the NF8 strain was constructed as follows. For segment A assembly, two overlapping fragments were generated through multi-step overlap PCR. The first fragment underwent three successive amplifications using primer pairs A1/A4, A2/A4, and A3/A4. The second fragment was amplified through four sequential PCR steps with primer pairs A5/A6, A5/A7, A5/A8, and A5/A9. Following purification, the full-length segment A flanked by hammerhead ribozyme (HamRz) and hepatitis delta virus ribozyme (HdvRz) sequences was generated via overlap PCR using primers A3/A9. The engineered fragment was digested with NotI/NheI and directionally cloned into pcDNA3.1 to generate infectious clone pc-NF8A. Based on the plasmid pc-NF8A, primer pairs A3/attIBDV-Q221K-R and attIBDV-Q221K-F/A9 were introduced Q221K mutation and the mutated plasmid was named pc-NF8A-221K. For segment B construction, two overlapping fragments were similarly amplified: the first using three-step PCR (B1/B4, B2/B4, B3/B4) and the second through four-step PCR (B5/B6, B5/B7, B5/B8, B5/B9). Full-length segment B was assembled using primers B3/B9. The fragment was digested with NotI/NheI and cloned into pcDNA3.1 to generate pc-NF8B. The infectious viruses rNF8 and its variant rNF8-221K were generated through co-transfection of LMH cells with either pc-NF8A or pc-NF8A-221K alongside pc-NF8B. At 5 days post-transfection (dpt), transfected cells underwent three freeze-thaw cycles to release viral progeny. The cell lysate was subsequently subcultured in fresh LMH cells for viral amplification. At 5 days post-inoculation (dpi), the infected cells were harvested for rescue virus confirmation via IFA and sequencing validation.

**Table 2:** PCR primers used for rescuing virus.

Primer	Direction	Primer sequence (5’ - 3’)
A1	Forward	GGATACGATCGGTCTGACCCCGGGGGAGTC
A2	Forward	TGAGGACGAAACTATAGGAAAGGAATTCCTATAGTCGGATACGATCGGTCTGAC
A3	Forward	ATAAGAATGCGGCCGCTGTTAAGCGTCTGATGAGTCCGTGAGGACGAAACTATAGGAAAG
A4	Reverse	TCTTTGATATCCGTGTGTCTTTTTCC
A5	Forward	CACGGATATCAAAGAAGATGGAGAC
A6	Reverse	GGGGACCCGCGAACGGATCCAATTTGGGAT
A7	Reverse	CGGACCGCGAGGAGGTGGAGATGCCATGCCGACCCGGGGACCCGCGAACGGATC
A8	Reverse	GAGTGGACGTGCGTCCTCCTTCGGATGCCCAGGTCGGACCGCGAGGAGGTGGAG
A9	Reverse	CTAGCTAGCCGCCCTCCCTTAGCCATCCGAGTGGACGTGCGTCCTCCTTC
B1	Forward	GGATACGATGGGTCTGACCCTCTGGGA
B2	Forward	TGAGGACGAAACTATAGGAAAGGAATTCCTATAGTCGGATACGATGGGTCTGAC
B3	Forward	ATAAGAATGCGGCCGCTGTTAAGCGTCTGATGAGTCCGTGAGGACGAAACTATAGGAAAG
B4	Reverse	ACTGCGTCCTGCAGACGGCTCCTTG
B5	Forward	CGTCTGCAGGACGCAGTTAAGGCCAAG
B6	Reverse	GGGGGCCCCCGCAGGCGAAGGCCGGGGAT
B7	Reverse	CGGACCGCGAGGAGGTGGAGATGCCATGCCGACCCGGGGGCCCCCGCAGGCGAAG
B8	Reverse	GAGTGGACGTGCGTCCTCCTTCGGATGCCCAGGTCGGACCGCGAGGAGGTGGAG
B9	Reverse	CTAGCTAGCCGCCCTCCCTTAGCCATCCGAGTGGACGTGCGTCCTCCTTC

### 2.10 Immunoprecipitation

LMH cells transfected with pc-nVarIBDV-VP2 or infected with pc-rNF8-221K were separately lysed in NP-40 lysis buffer for 30 min on ice. Following centrifugation at 12,000 × g for 10 min at 4°C, the resultant supernatant was incubated with the specified antibodies overnight and followed by an addition of Protein A/G magnetic beads for 3 h at 4°C. Upon centrifugation at 1,000 × g for 5 min at 4°C, the supernatant was discarded, and the beads were washed five times using ice-cold PBS. Then, the beads were lysed in SDS loading buffer and subjected to Western blot assays.

### 2.11 Neutralization assay

MAbs 5B5 and 6C5 were diluted to 60 µg/mL each. The mutant rNF8-221K was diluted to 1000 TCID50 per 500 µL and mixed with equal volumes of the diluted mAbs or nVarIBDV antiserum. After incubation at 37°C for 1 h, 1 mL of the mixture was added to each well of a 6-well plate containing a 90% confluent LMH cell monolayer. At 2 hours post-infection (hpi), the cells were divided into two experimental groups: one group was maintained in DMEM/F12 maintenance medium containing 1% FBS, while the other group received additional supplementation with the mAbs 5B5 or 6C5 (6 µg/mL) or nVarIBDV antiserum in the maintenance medium. Cells from both groups were harvested at 4 days post-infection (dpi) for Western blot analysis to assess viral replication. In a parallel experiment, LMH cells were first infected with rNF8-221K for 2 h and subsequently cultured in DMEM/F12 medium supplemented with 1% FBS and mAbs 5B5 or 6C5 concentration gradients. Supernatants were collected at 24-hour intervals, and at 4 dpi, cells were collected for Western blot analysis, while the corresponding supernatants were subjected to TCID50 assays.

### 2.12 VP2-HVR sequence and spatial structure analysis

The VP2-HVR amino acid sequences of IBDVs circulating in China, including nVarIBDV, vvIBDV, cIBDV, varIBDV and attIBDV, were retrieved from GenBank (listed in **Table 3**), and were aligned in DNAstar MegAlign software. The tertiary structure of VP2 protein was gathered from the Protein Data Bank (PDB; http://www.rcsb.org/pdb/). The PyMOL software (Version 2.5.2, Schrödinger, LLC) was used to visualize the spatial distribution of epitopes recognized mAb 5B5.

**Table 3:** Reference sequences used for the homology alignment of hypervariable region.

Strain	Phenotype	Genotype	Genbank No.
IBDV-JS19-13203	nVarIBDV	A2d	MT087551
SHG19			MH879045
SHG120			MH879063
SHG308			MH879122
IBDV-JS19-13202			MT087550
QZ191002			MZ066613
ZD-2018-1			MN485882
SHG326			MH879126
SHG115			MH879109
SHG475			MN218112
BX			AF413070
GZ902			AF006699
Variant E	varIBDV	A2a	AF133904
FW2512	cIBDV	A1	DQ656499
NN040124			DQ656502
IBD17JL012			MN604241
IBDV-GD19-15005			MW682890
NN0704	vvIBDV	A3	FJ615511
YS07			FJ695138
B-SD-RZ			GQ166972
SD10LY01			KF569803
HuB-1			KF569805
Gx		A3	AY444873
S18			MK472711
QL			JX682709
GL1001			KC968831
HLJ0504			GQ451330
Gt	attIBDV	A8	DQ403248
BH15			DQ656498
JD1			AF321055
HuN0804			FJ615498
QX110603			KC918849

### 2.13 Statistical analysis

All data were shown as the means ± SD of three independent experiments. Statistical differences were assessed using Student’s t- test and an unpaired two- tailed Student’s t- test using GraphPad Prism V.7.0 software. For all experiments, p < 0.05 was considered statistically significant (*p<0.05, **p<0.01).

## 3 Results

### 3.1 Expression and purification of the recombinant VP2-HVR protein

**Figure 2:**
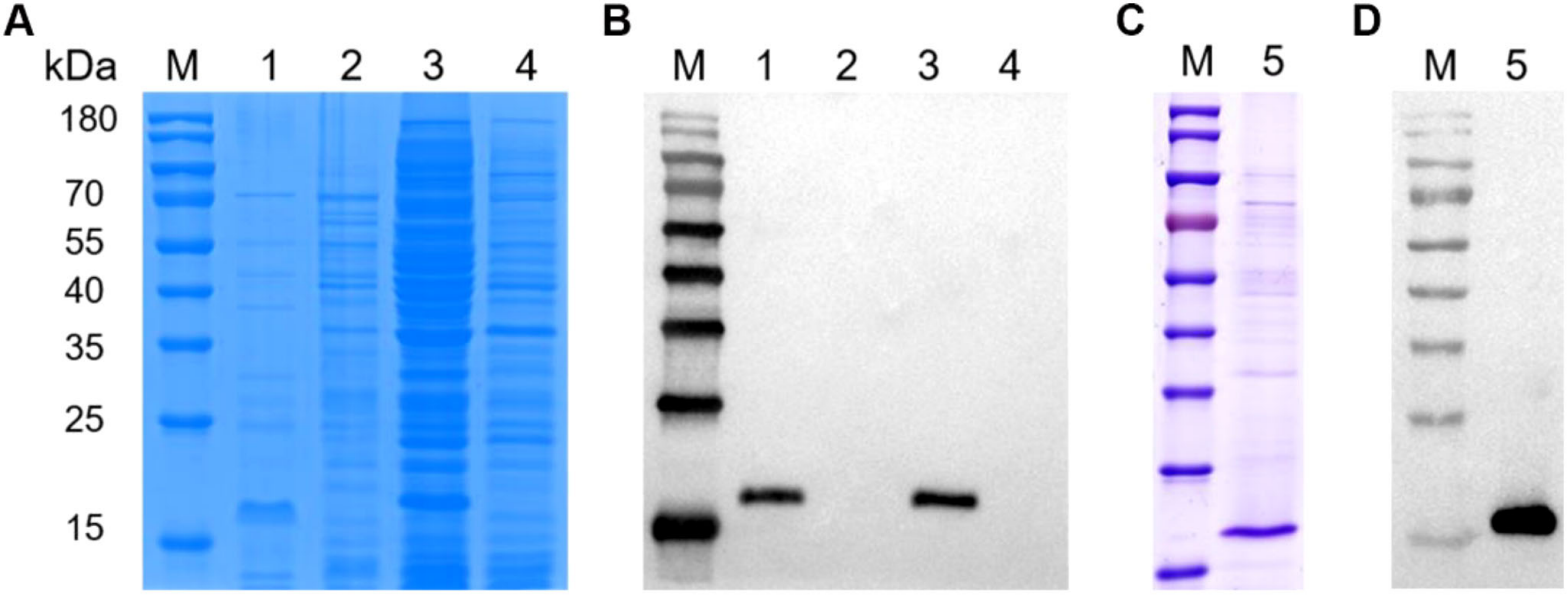
Recombinant VP2-HVR protein was water-soluble. Recombinant plasmid pCold-I-VP2-HVR or empty plasmid pColdI was transformed into E. coli Rosetta (DE3) cells, followed by induction with 1.0 mM IPTG at 16°C for 15 h. **(A, C)** SDS-PAGE analysis for the expression and purification of the recombinant VP2-HVR protein in E. coli. **(B, D)** Western blot analysis for the expression and purification of the recombinant VP2-HVR protein in E.coli. Lane M, Protein Marker; Lane 1 and 3, the supernatant and the precipitation of the lysate of E. coli Rosetta (DE3) transformed with pColdI-VP2-HVR, respectively. Lane 2 and 4, the supernatant and the precipitation of the lysate of E. coli Rosetta (DE3) transformed with pColdI, respectively. Lane 5, purified VP2-HVR protein.

To obtain the VP2-HVR protein, the recombinant plasmid pColdI-VP2-HVR was first constructed, and then transformed into the *E. coli* Rosetta cells and expressed as His-tagged fusion protein. As shown in **Figure 2A**, The recombinant VP2-HVR protein was successfully expressed in a soluble form in the Rosetta (DE3) expression system. SDS-PAGE analysis revealed a specific band of approximately 18 kDa in the supernatant, which corresponded to the expected molecular weight. In contrast, no corresponding band was detected at this molecular weight range in the empty pColdI vector control group. This expression was further confirmed by an anti-His monoclonal antibody (**Figure 2B**). Next, the recombinant VP2-HVR protein was purified using a Ni Sepharose™ affinity chromatography column. As shown in **Figure 2C**, SDS-PAGE revealed a specific band corresponding to the recombinant VP2-HVR protein. Furthermore, Western blot assay indicated that the recombinant VP2-HVR protein could be effectively recognized by chicken sera against nVarIBDV (**Figure 2D**). Collectively, these results demonstrated that the prokaryotic recombinant VP2-HVR protein of nVarIBDV was successfully expressed with good antigenicity.

### 3.2 Generation of a novel mAb against VP2 of nVarIBDV

**Figure 3:**
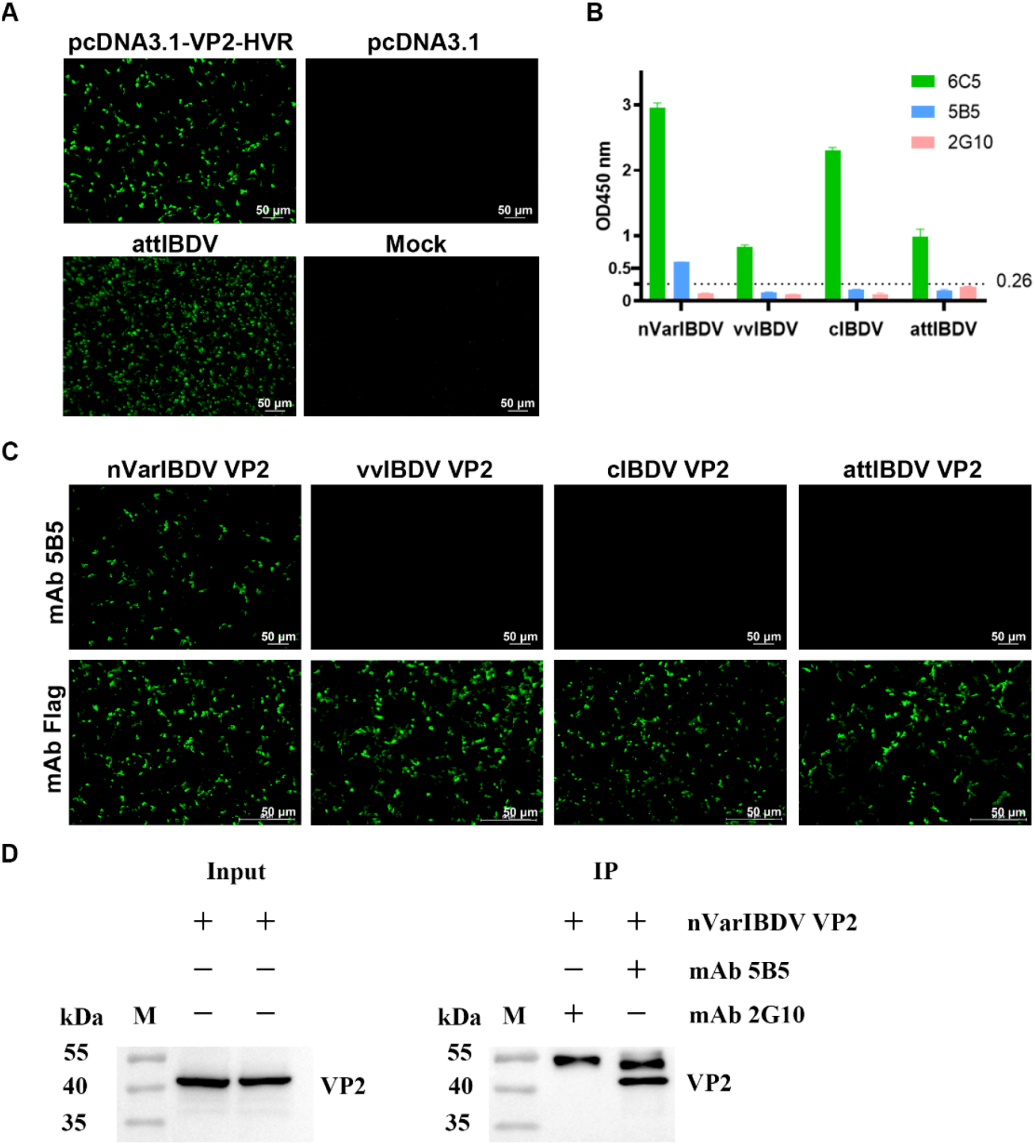
MAb 5B5 specifically recognizes nVarIBDV and its VP2 protein. Mice were immunized with recombinant VP2-HVR protein, and the mouse showing the highest anti-VP2 serum titers was selected for monoclonal antibody production. **(A)** IFA identification of VP2-HVR protein and attIBDV with anti-IBDV VP2 polyclonal antiserum at 48 h after transfection or infection of LMH cells. **(B)** Sandwich ELISA analysis of the reactivity of mAb 5B5 with different types of IBDV (nVarIBDV, vvIBDV, cIBDV and attIBDV). Broadly reactive mAb 6C5 served as capture antibody, with HRP-conjugated mAb 5B5, 6C5, or 2G10 (isotype control) as detection antibodies. Error bars represent the SD. **(C)** IFA of mAb 5B5 against VP2 proteins from diverse IBDV strains in LMH cells at 48 h post-transfection. **(D)** Immunoprecipitation assay of VP2 protein in LMH cells transfected with pc-nVarIBDV-VP2 at 48 h post-transfection, using mAbs 5B5 and 2G10 (negative control).

To generate monoclonal antibodies (mAbs) targeting the VP2 protein of nVarIBDV, BALB/c mice were immunized with purified recombinant VP2- HVR protein derived from nVarIBDV. Since only an attIBDV strain (NF8) - but not nVarIBDV, vvIBDV, or cIBDV - was adapted to cell culture in our study, the positive sera from immunized mice were verified by reacting with LMH cells transfected with pc-VP2-HVR or infected with NF8. IFA results revealed strong serum reactivity against VP2-HVR and NF8 (**Figure 3A**). Splenocytes from high-titer responders were subsequently fused with SP2/0 myeloma cells, yielding 16 positive hybridoma clones. Among these, mAb 5B5 demonstrated strain-specific recognition of nVarIBDV, showing no cross-reactivity with vvIBDV, cIBDV or attIBDV strains in an in-house established IBDV-specific sandwich ELISA assay (**Figure 3B**). The cutoff value was calculated as 0.26 using the formula: cutoff = OD450 mean + 3×SD based on 43 avian-derived viral samples (OD450 mean = 0.086, SD = 0.058). To further confirm the specificity of mAb 5B5 for nVarIBDV, LMH cells transfected with plasmids pc-nVarIBDV-VP2, pc-vvIBDV-VP2, pc-attIBDV-VP2 and pc-cIBDV-VP2 were used to IFA and Western blot analysis. As described in **Figure 3C**, mAb 5B5 specifically recognized VP2 protein of nVarIBDV, but not that of vvIBDV, cIBDV or attIBDV strains, which was consistent with the data of ELISA. However, 5B5 could not react with any denatured VP2 protein of different IBDV types including nVarIBDV, vvIBDV, cIBDV or attIBDV (data not shown), suggesting that mAb 5B5 recognized the conformational epitope of VP2. To validate the binding specificity of monoclonal antibody 5B5 to nVarIBDV VP2, immunoprecipitation assays were conducted in LMH cells transiently expressing VP2 proteins from nVarIBDV, vvIBDV, cIBDV, and attIBDV. As shown in **Figure 3D**, mAb 5B5 demonstrated strong binding affinity for nVarIBDV VP2, while showed no detectable binding for VP2 proteins of vvIBDV, cIBDV or attIBDV (data not shown). Collectively, these data demonstrate that mAb 5B5 specifically recognizes nVarIBDV-derived VP2 without cross-reactivity to VP2 from other IBDV phenotypes.

### 3.3 MAb 5B5 recognized 221K in VP2 of nVarIBDV

**Figure 4:**
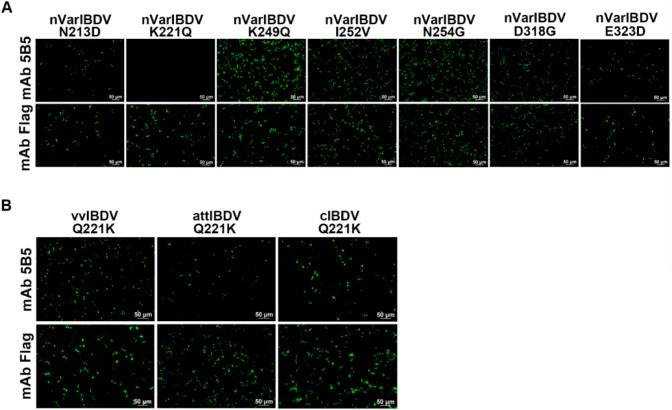
MAb 5B5 recognizes the crucial residue 221K of VP2. N213D, K221Q, K249Q, I252V, N254G, D318G, or E323D mutation was individually introduced into the VP2 of nVarIBDV while the compensatory mutation Q221K was engineered into VP2 proteins of vvIBDV, cIBDV, and attIBDV for crucial epitope mapping. **(A, B)** IFA identification of the crucial residue recognized by mAb 5B5.

To localize the conformational B-cell epitope recognized by mAb 5B5, a series of truncated VP2-HVR constructs were designed (**Figure 1A**). In the first round, five truncated fragments of the VP2-HVR protein from nVarIBDV were generated and used to map the epitope recognized by mAb 5B5. As described in **Figures 1B, C**, mAb 5B5 reacted only with the V1 (201-347aa) fragment, but not with the other constructed fragments. In the second round, V1 was further truncated to precisely define the epitope recognized by mAb 5B5. As shown in **Figures 1C, D**, mAb 5B5 reacted only with V6 (201-337aa). Notably, the deletion of the peak A (DNYQFSSQYKTPGG, 212-225aa) or the heptapeptide region (WSASGSS, 326-332aa) of the VP2 protein from nVarIBDV abolished the reaction between mAb 5B5 and the VP2 protein. Since there are seven amino acid differences (including D213N, Q221K, Q249K, V252I, G254N, G318D, and D323E) in the peak A and the heptapeptide region of VP2-HVR between nVarIBDV and other IBDV strains, to further determine the key residues recognized by mAb 5B5, N213D, K221Q, K249Q, I252V, N254G, D318G, or E323D mutation was individually introduced into the VP2 of nVarIBDV. The corresponding plasmids, designated as pc-nVarIBDV-N213D, pc-nVarIBDV-K221Q, pc-nVarIBDV-K249Q, pc-nVarIBDV-I252V, pc-nVarIBDV-N254G, pc-nVarIBDV-D318G, and pc-nVarIBDV-E323D, were subsequently generated. As described in **Figure 4A**, IFA analysis revealed that mAb 5B5 could still react with the VP2 of nVarIBDV carrying N213D, K249Q, I252V, N254G, D318G or E323D, but not with the VP2 of nVarIBDV with K221Q. To further confirm this, a panel of VP2 mutant of vvIBDV, cIBDV, or attIBDV with Q221K were constructed. As shown in **Figure 4B**, mAb 5B5 could effectively recognize the VP2 of vvIBDV, cIBDV, or attIBDV with Q221K. All these data demonstrated that the epitope recognized by mAb 5B5 is located in the peak A and heptapeptide regions of VP2, and 221K in VP2 of nVarIBDV is the key antigenic site in the epitope for mAb 5B5.

### 3.4 Q221K mutation altered antigenicity of VP2

**Figure 5:**
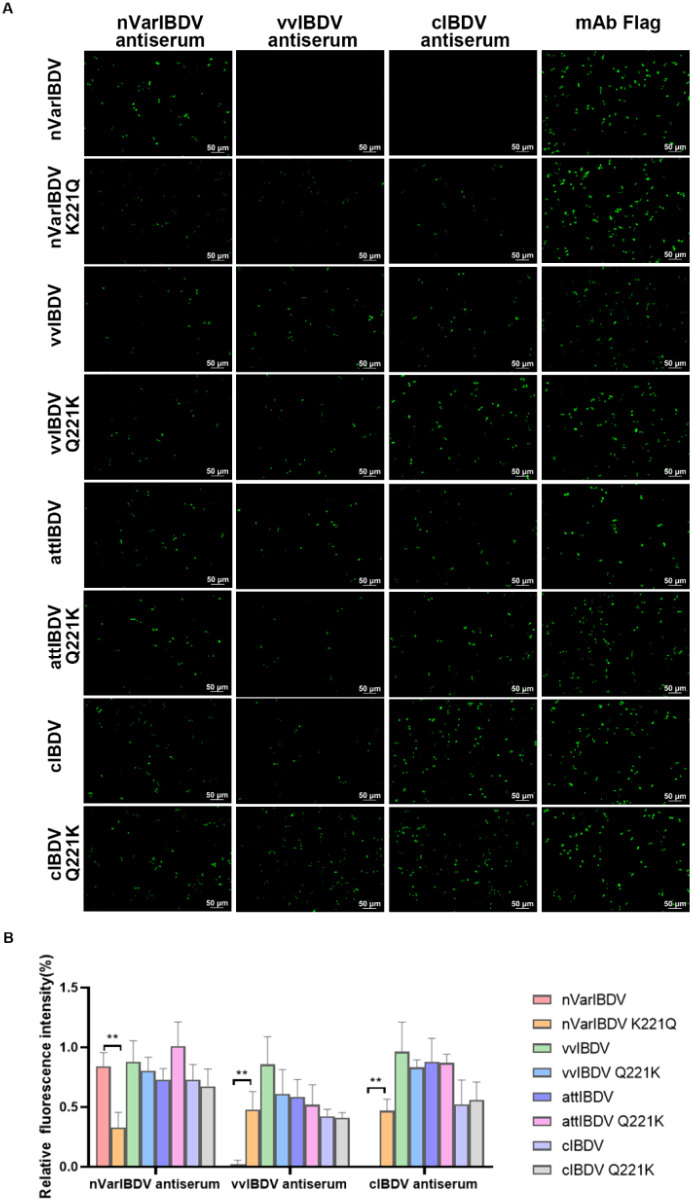
Q221K mutation altered antigenicity of VP2. Wild-type VP2 proteins of nVarIBDV, vvIBDV, cIBDV, and attIBDV, along with their corresponding VP2 variants carrying K221Q or Q221K substitutions, were transfected into LMH cells. At 48 hpt, **(A)** IFA analysis of nVarIBDV-, vvIBDV-, and cIBDV-specific antisera binding to wild-type VP2 proteins and their K221Q/Q221K mutants. **(B)** Quantification of fluorescence signals corresponding to **(A)**. The percentage of nVarIBDV-, vvIBDV-, and cIBDV-bound positive cells (green fluorescence) to mAb Flag-bound positive cells (green fluorescence) was showed by using ImageJ (NIH). **p<0.01.

To further investigate the effect of the Q221K mutation on the antigenicity of VP2, LMH cells were transfected with wild-type VP2 of nVarIBDV, vvIBDV, cIBDV, and attIBDV, along with their corresponding mutants with Q221K or K221Q, and then were analyzed by IFA using chicken sera against different types of IBDV. As described in **Figure 5**, although sera against nVarIBDV could recognize all the wild type VP2 proteins and their corresponding mutants, it had significantly enhanced reactivity with the wild type of VP2 of nVarIBDV compared to its mutant with K221Q. Notably, sera against vvIBDV or cIBDV demonstrated favorable affinity for the VP2 proteins of vvIBDV, cIBDV, and attIBDV, as well as their respective mutants. However, sera against vvIBDV or cIBDV could not recognize the wild type VP2 protein of nVarIBDV, but could react with the VP2 of nVarIBDV with K221Q (**Figure 5**). All these demonstrated that Q221K mutation not only serves as key antigenic site recognized by mAb 5B5, but also significantly alters the antigenicity of VP2 of nVarIBDV, which might be associated with the immune escape of nVarIBDV from current IBDV vaccine.

### 3.5 MAb 5B5 could neutralize IBDV in LMH cells

**Figure 6:**
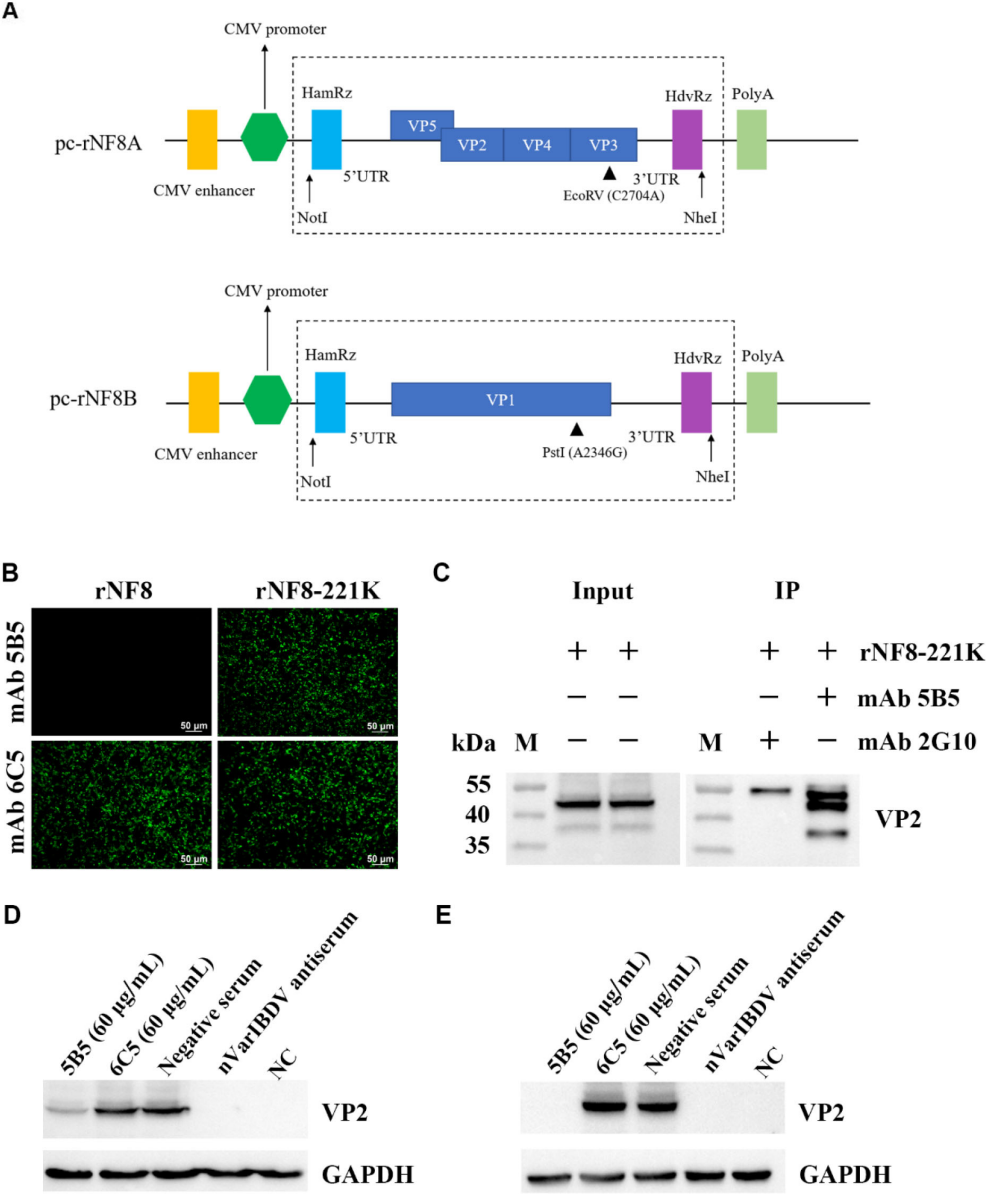
MAb 5B5 efficiently inhibited the infection of IBDV *in vitro*. **(A)** Schematic representation of pcDNA3.1 expression plasmids carrying genome segments A and B from the attenuated IBDV strain NF8. **(B)** IFA identification of the wild-type rNF8 and mutant rNF8-221K viruses with mAb 5B5 at 48 h post-infection of LMH cells. **(C)** Immunoprecipitation assay of rNF8-221K mutant virus using mAb 5B5 and control mAb 2G10. MAb 5B5 (60 μg/mL) was mixed with 100 TCID_50_ viral suspension at a 1:1 volume ratio, neutralized at 37°C for 1 (h) Following 2 h adsorption on LMH cells, the inoculum was replaced with maintenance medium containing fresh antibody (60 μg/mL) or control medium. Neutralization activity of mAb 5B5 was assessed by Western blot at 4 day post-infection. **(D)** Neutralization assay of mAb 5B5 against rNF8-221K in the absence of mAb 5B5 in the culture medium. **(E)** Neutralization test of mAb 5B5 (60 μg/mL) against mutant virus rNF8-221K in the culture medium.

**Figure 7:**
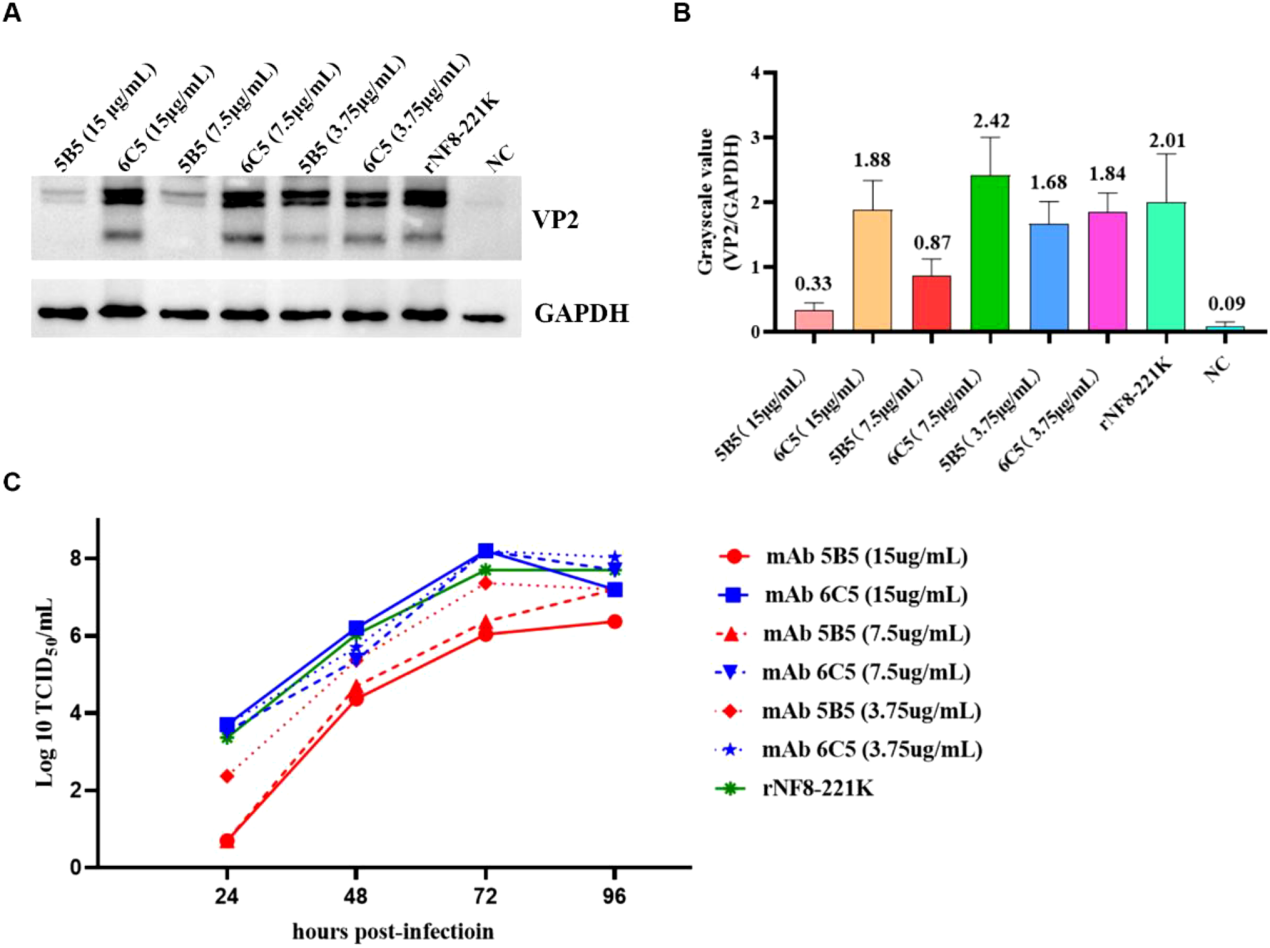
MAb 5B5 inhibits IBDV egress. Serial dilutions of mAb 5B5 (0-15 μg/mL) was mixed with 100 TCID_50_ viral suspension, neutralized at 37°C for 1 h, then inoculated onto LMH cells. After 2 h incubation, the virus-antibody suspension was removed, and maintenance medium containing corresponding concentrations of mAb 5B5 was added for continued culture. Residual viral titers were determined at 4 days post-infection through Western blot **(A)** and TCID_50_ **(C)** assays. **(B)** Grayscale analysis of **(A)** panel using ImageJ (NIH) by measuring the optical density (OD) of regions of interest (ROIs). Error bars represent the SD. For **(B, C)** panels images were performed in GraphPad Prism V.7.0.

To assess the inhibitory potential of monoclonal antibody 5B5 (mAb 5B5) on IBDV infectivity, we employed reverse genetics to rescue a point-mutated attenuated strain, designated rNF8-221K (**Figure 6A**), owing to the limited permissiveness of nVarIBDV in LMH, DF-1, and DT40 cell lines. The rescued strain was validated by IFA and Sanger sequencing (data not shown). Notably, mAb 5B5 exhibited specific binding to rNF8-221K but not to the parental strain rNF8 (**Figure 6B**). Moreover, a high-affinity interaction between mAb 5B5 and rNF8-221K was further confirmed by in immunoprecipitation (**Figure 6C**). Neutralization assays demonstrated that supplementation with 60 μg/mL mAb 5B5 in the culture medium significantly suppressed rNF8-221K infection/replication in LMH cells compared to untreated controls (**Figures 6D, E**). To investigate the potential inhibitory effect of mAb 5B5 on viral egress, rNF8-221K-infected LMH cells were treated with varying concentrations of mAb 5B5 or control mAb 6C5. Western blot and TCID_50_ analyses demonstrated that mAb 5B5 significantly suppressed viral replication in a dose-dependent manner, with marked inhibition observed even at 7.5 μg/mL (**Figures 7A–C**). In contrast, the control mAb 6C5 showed no comparable inhibitory activity. These findings indicated that mAb 5B5 effectively restricts IBDV replication during the intracellular phase of infection.

### 3.6 221K situated at the outermost tip of the peak A region

**Figure 8:**
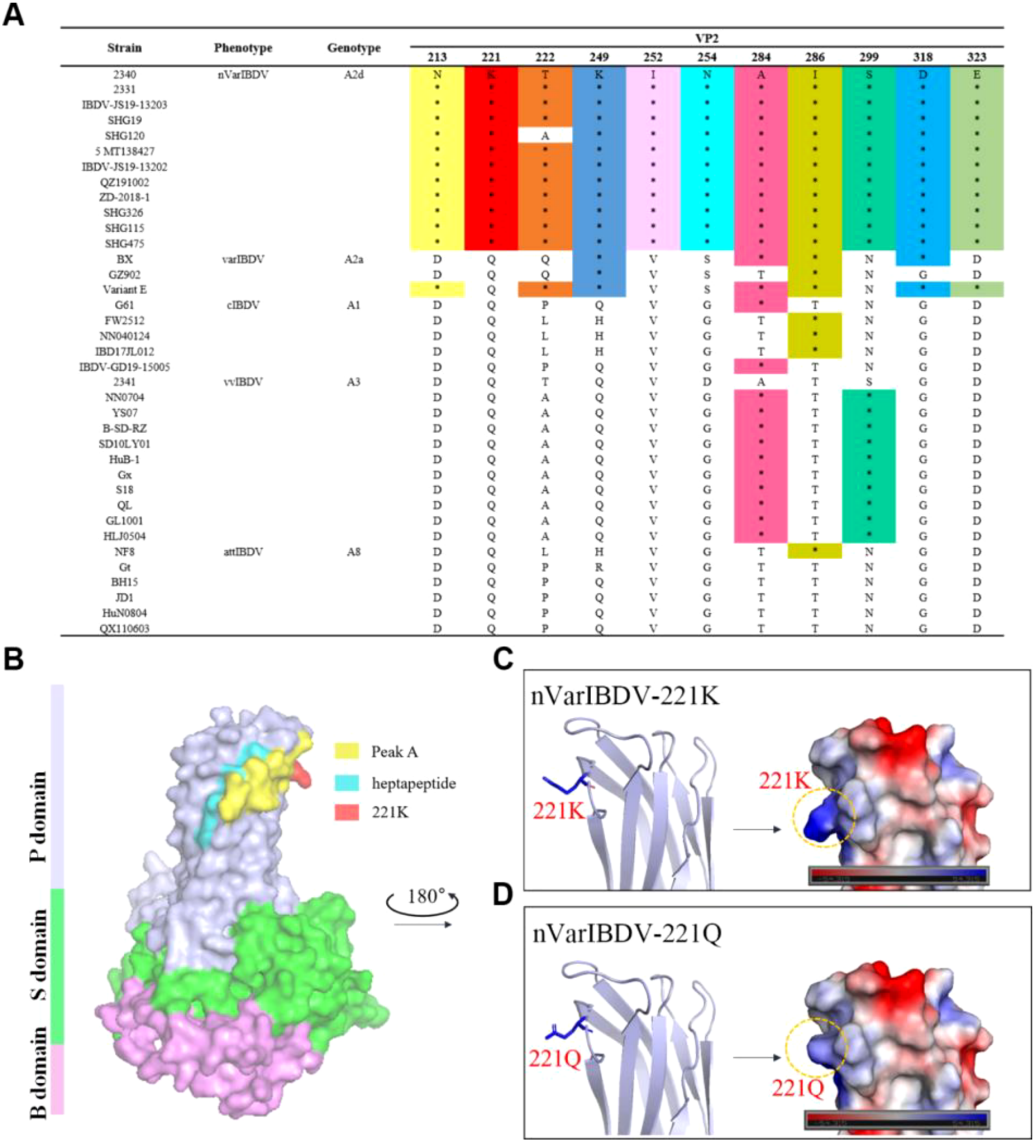
Q221K mutation perturbs localized surface electrostatic potential of VP2. **(A)** Unique amino-residue markers of nVarIBDV. **(B)** Overall 3D structure of nVarIBDV VP2. P, S, and B domains were marked with different colors. The antigen epitopes (peak A and heptapeptide region) and key residue 221K were highlighted. **(C)** A magnified local P - region view of nVarIBDV-221K. **(D)** a magnified local P - region view of nVarIBDV-221Q. The surface potential energy of residue 221 is marked with a dashed box.

To assess sequence conservation of the key 221K antigenic site within VP2 (recognized by mAb 5B5), we aligned representative VP2 sequences from major IBDV types circulating in China—including nVarIBDV, varIBDV, vvIBDV, cIBDV, and attIBDV—alongside five laboratory-isolated strains. The results identified 11 unique amino acid residues within the VP2-HVR: 213N, 221K, 222T, 249K, 252I, 254N, 284A, 286I, 299S, 318D, and 323E. Among these, 213N, 221K, 252I, 254N, 299S, 318D, and 323E served as significant molecular markers distinguishing Chinese nVarIBDV (A2d) from the Chinese isolates of A2a type variants BX (1996), GZ901 (1996) and early U.S. varIBDV strains Variant E. Furthermore, 221K and 252I were exclusively present in Chinese nVarIBDV (A2d), constituting distinguishing features from other IBDV genotypes (**Figure 8A**). The spatial structure of the peak A region and the heptapeptide region in the VP2 protein was visualized using PyMOL software. As described in **Figure 8B**, the peak A region and the heptapeptide region were exposed on the surface of the P domain of the VP2 protein and were spatially adjacent to each other. This distribution contributed to the formation of the conformational epitope recognized by mAb 5B5. Moreover, the key antigenic site 221K was situated at the outermost tip of the peak A, making it more spatially accessible for binding with antibodies. Notably, although the substitution of residue 221 from Q to K did not change the overall structure of VP2 (**Figures 8C, D**), it altered local electrostatic potential, possibly facilitating antigenicity and immune evasion.

## 4 Discussion

Over the past three decades, vvIBDV has dominated in China, exhibiting stronger pathogenicity than cIBDV (40) while maintaining antigenic similarity (41). Current control primarily relies on live attenuated vaccines developed by blind passage of vvIBDV and moderate virulence cIBDV-based vaccines, with emerging alternatives including subunit and genetic engineering vaccines (42–45). Notably, a novel variant (nVarIBDV) emerged in 2016 (23), phylogenetically linked to the American antigenic variant IBDV (varIBDV) introduced in the 1990s (23, 46). While retaining characteristic varIBDV residues (222T/249K/286I/318D) in the VP2 hypervariable region, nVarIBDV acquired three distinct mutations (221K/252I/299S) serving as molecular identifiers (23). Crucially, nVarIBDV demonstrates significant antigenic divergence from vvIBDV (35, 47, 48), rendering existing vaccines ineffective and driving escalating infection rates in Chinese poultry populations (20, 34).

To preliminarily screen the basis of molecular variation in nVarIBDV, we generated a specific monoclonal antibody (mAb 5B5) using prokaryotically expressed VP2-HVR protein of nVarIBDV as the immunogen. This antibody exclusively reacts with nVarIBDV VP2 protein without cross-reactivity to other IBDV subtypes (**Figure 3**). Epitope mapping revealed that mAb 5B5 recognizes both hydrophilic peak A and the heptapeptide region of VP2 (**Figure 1**). Although these two domains are spatially distant in the primary protein structure, they form adjacent structural motifs in modeling analysis of the tertiary structure of VP2 (**Figure 8B**). Notably, mAb 5B5 failed to react with any denatured VP2 protein in Western blot assays (data not shown), collectively demonstrating its recognition of conformational epitopes in VP2. In comparison with the sequence of VP2-HVR from different types of IBDV, we found that residues 213N, 221K, 249K, 252I, 254N, 318D and 323G of VP2 of nVarIBDV were distinct from other non-variant IBDV strains. Site mutagenesis assay revealed that 221K in VP2 of nVarIBDV was the key antigenic site in the epitope recognized by mAb 5B5 (**Figure 4A**). Notably, 221K is highly invariant in VP2 of nVarIBDV, whereas the VP2 from other types of IBDV carries 221Q (**Figure 8A**). Further mutagenesis analysis showed mAb 5B5 could efficiently react with VP2 of vvIBDV, cIBDV and attIBDV with Q221K mutation, but not with the wild type VP2 with 221Q (**Figure 4B**). Moreover, we found that the 221K site in VP2 of nVarIBDV, located on the exposed surface of the P_BC_ loop (a region affecting antigen-antibody affinity) (35), may directly regulate antibody recognition.

To validate this hypothesis, we conducted IFA to assess the reactivity of three chicken antisera (nVarIBDV, vvIBDV and cIBDV) against VP2 proteins of nVarIBDV, vvIBDV, cIBDV, and attIBDV, as well as their variant VP2 proteins carrying 221Q or 221K. The results showed that sera against vvIBDV or cIBDV could not react with the wild type VP2 protein of nVarIBDV with 221K, but could efficiently react with the VP2 of nVarIBDV with 221Q (**Figure 5**). This highlights that Q221K mutation not only significantly alters the antigenicity of VP2, but also contributes the immune escape of nVarIBDV from current IBDV vaccine. Sequence alignment of diverse IBDV genotypes circulating in China revealed that nVarIBDV (A2d) possesses 11 distinctive amino acid markers, among which 221K and 252I serve as signature residues significantly differentiating it from other IBDV genotypes (**Figure 8A**). It is widely accepted that nVarIBDV originated from early US varIBDV (23, 46). Comparative selection pressure analysis of IBDVs during 1985–2015 (endemic period of early US varIBDV) and 2016–2021 (epidemic period of Chinese nVarIBDV) identified strong positive selection at codons 212, 213, 221, 222, 254, 284, and 318 during 1985–2015 (46). These codons are located within hydrophilic loops of the VP2 P-domain—regions critically associated with IBDV antigenicity. In addition, a novel IBDV genotype (A9B1) reported in Portugal harbors a unique K221-S222 amino acid pattern within the VP2 hypervariable region (31). These findings indicate IBDV’s evolutionary strategy of immune escape through mutations in conventional antigenic sites, consistent with our finding that Q221K mediates antigenic drift in nVarIBDV. Furthermore, Post-2016, three new positively selected sites emerged in nVarIBDV: codon 209 (adjacent to loop P_BC_) and codons 347/349 (adjacent to loop P_HI_), both implicated in viral antigenicity, which suggests nVarIBDV may undergo future adaptive mutations at positions 209, 347, and 349 in response to environmental selection pressures. Structural modeling further showed that the Q to K substitution introduces a positively charged lysine at position 221, altering the local electrostatic potential without perturbing overall conformation structure of VP2 (**Figure 8C**). This charge reversal likely disrupts electrostatic complementarity between vaccine-induced antibodies and viral epitopes, thereby potentially explaining the nVarIBDV strain’s ability to evade pre-existing vaccine-induced immunity. Notably, similar charge-driven antigenic shifts have been reported in influenza viruses (49), suggesting a conserved evolutionary strategy among RNA viruses. In addition, according to the previous reports, residues 1-40aa, 197-209aa, 210-225aa, 220-240aa, 329-337aa, 314-324aa, 320-340aa, 380-400aa and 400-420aa in VP2 of IBDV contain neutralizing epitopes (50).

To determine whether the monoclonal antibody 5B5 exhibits neutralizing activity against IBDV, we conducted neutralization assays. Notably, in this study, nVarIBDV showed no infectivity toward DF-1, DT40 or LMH cell lines. Despite multiple attempts, we were unable to serially passage or rescue the nVarIBDV strain in these cells. Ultimately, only the rNF8 (attIBDV) strain and its point-mutant variant rNF8-221K were successfully rescued in LMH cells, whereas rescue attempts in DT40 or DF-1 cells were largely unsuccessful. This limitation may be related to the low transfection efficiency observed in DF-1 and DT40 cells under our experimental conditions. Additionally, LMH cells exhibit stronger foreign gene protein expression than DF-1 cells (51). Although the rescued IBDV infects both LMH and DF-1 cells, LMH cells appear more permissive, with viral titers reaching up to 10^8^ to 10^9^ TCID50/mL (data not shown). In the neutralization assay, we found that mAb 5B5 showed efficiently neutralizing activity against rNF8-221K in LMH cells, highlighting the key antigenic site 221K recognized by mAb 5B5 plays vital roles in inducing neutralizing antibody against nVarIBDV. Conventional neutralization assays demonstrated that high-dose mAb 5B5 failed to completely neutralize IBDV infection (**Figure 6**). However, post-adsorption administration of 7.5 μg/mL 5B5 in cell culture systems significantly inhibited the replication kinetics of IBDV (**Figure 7A**). This observation suggests that, in addition to direct neutralization, mAb 5B5 may exert antiviral effects through non-neutralizing mechanisms targeting later stages of the viral life cycle. As the primary structural component of the IBDV capsid, VP2 requires precise conformational folding for proper capsid assembly and virion stability (52). The trimeric configuration of VP2 molecules forms surface projections critical for host cell attachment and viral entry, while its hypervariable region drives antigenic variation and immune escape. MAb 5B5 binding to VP2 may induce conformational changes or occlude critical epitopes within these functional domains, potentially disrupting capsid assembly, reducing virion stability, or interfering with progeny virion release. Notably, VP2 interacts dynamically with host cellular components during infection. The IBDV replication cycle involves VP2-mediated recruitment of Golgi-resident enzyme CSGalNAcT2, which facilitates viral replication through mechanisms dependent on Golgi integrity (53). Furthermore, cytoskeletal components such as vimentin and α-tubulin participate in intracellular trafficking of viral components (54). MAb 5B5 binding might perturb these VP2-mediated interactions, potentially hindering viral transport to replication sites or assembly compartments. The dual mechanisms – structural interference with capsid assembly and functional disruption of VP2-host interactions – warrant further exploration.

## 5 Conclusion

This is the first identification of a novel antigenic site 221K in VP2 of nVarIBDV recognized by mAb 5B5 with neutralizing activity, opening new avenues for combating nVarIBDV. First, the residue 221K could serve as a molecular marker for rapid differentiation of nVarIBDV from other types of IBDV, enhancing diagnostic accuracy. Second, incorporating 221K into vaccine formulations may improve cross-protection. To validate this, *in vivo* studies using 221K-engineered live-attenuated viruses are warranted. Additionally, exploring synergistic effects between 221K and neighboring residues (e.g., 318D, 323G) could elucidate combinatorial impacts on antigenicity. Finally, investigating whether 221K influences viral replication or virulence via reverse genetics technology would deepen our understanding of nVarIBDV pathogenesis.

## Data Availability

The original contributions presented in the study are included in the article/supplementary material. Further inquiries can be directed to the corresponding authors.
